# The Absence of NLRP3-inflammasome Modulates Hepatic Fibrosis Progression, Lipid Metabolism, and Inflammation in KO NLRP3 Mice during Aging

**DOI:** 10.3390/cells9102148

**Published:** 2020-09-23

**Authors:** Paloma Gallego, Beatriz Castejón-Vega, José A. del Campo, Mario D. Cordero

**Affiliations:** 1 Unit for Clinical Management of Digestive Diseases and CIBERehd, Valme University Hospital, 41014 Seville, Spain; palgalyer@alum.us.es; 2 Research Laboratory, Oral Medicine Department, University of Sevilla, 41009 Sevilla, Spain; beacastej92@outlook.es; 3 AlgaEnergy S.A. Avda. Europa 19, 28108 Alcobendas, Madrid, Spain; 4 Cátedra de Reproducción y Genética Humana del Instituto para el Estudio de la Biología de la Reproducción Humana (INEBIR), Universidad Europea del Atlántico (UNEATLANTICO), Fundación Universitaria Iberoamericana (FUNIBER), 41001 Sevilla, Spain

**Keywords:** non-alcoholic fatty liver disease, oxidative stress, liver damage, inflammation, NLRP3-inflammasome complex

## Abstract

Aging is associated with metabolic changes and low-grade inflammation in several organs, which may be due to NLRP3 inflammasome activation. Methods: Here, we asked whether age-related liver changes such as lipid metabolism and fibrosis are reduced in aged mice lacking the NLRP3 inflammasome. We report reduced protein levels of lipid markers (MTP, FASN, DGAT1), SOD activity, oxidative stress marker PTPRG, and the fibrotic markers TPM2β, COL1-α1 associated with increased GATA4, in NLRP3 deficient mice. Fibrotic, lipid, and oxidative reduction in liver tissues of mice was more pronounced in those old KO NLRP3 mice than in the younger ones, despite their greater liver damage. These results suggest that absence of the NLRP3 inflammasome attenuates age-related liver fibrotic pathology in mice, suggesting that pharmacological targeting may be beneficial.

## 1. Introduction

Hepatic inflammation is one of the liver disease triggering processes. Inflammation is considered the main factor involved in liver damage, since it enhances the progression from non alcoholic fatty liver disease (NAFLD) to severe fibrosis, until finally developing hepatocellular carcinoma [1]. Liver disease, whose pathogenesis is complex, is explained by an excessive lipid accumulation in the hepatocytes, added to an oxidative stress [2]. Damage by oxidative stress generates a lipotoxicity environment, which in turn triggers the inflammatory response, causing liver inflammation and fibrosis progression [3]. The release of saturated fatty acids and peroxidized lipids during cell damage are recognized as endogenous molecules by pattern recognition receptors (PPR), among which are the NOD-type receptors (NLR), with the NLRP3 inflammasome being the best characterized. The NLRP3 inflammasome, once it recognizes these molecules, is activated, and it can associate and form a complex with the apoptosis-associated speck-like protein to CARD (ASC), activating caspase-1 and inducing the maturation and secretion of proinflammatory cytokines such as IL-1B and IL-18 [4,5]. In this necroinflammatory environment, any of these mechanisms of oxidative stress and lipid accumulation could activate hepatic stellate cells (HSCs), facilitating fibrogenic progression [6]. There are different studies that ensure the relationship between the NLRP3 inflammasome complex and liver lesions, increasing the expression of liver NLRP3 and caspase 1, and finally generating an inflammatory process in the liver [7,8].

Throughout this molecular framework within liver disease and fibrosis, age could be identified as a factor for the severe progression of hepatic fibrosis. Aging is a process characterized by the progressive loss of tissue and organic function due to the accumulation of reactive oxygen species [9]. During aging, many alterations occur in the organism, which are associated with progressive impairment of metabolic pathways [10]. A deterioration of liver function associated with age is frequent, in addition to the appearance of NASH, which can progress to liver fibrosis, cirrhosis, and hepatocarcinoma, aggravating the liver inflammatory state [11]. The NLRP3 inflammasome complex can be overactivated, producing an aggravated inflammatory process, after chronic damage, such as metabolic disturbances that occur during NAFLD and liver fibrosis, or during a process of progressive deterioration associated with aging. According to these data, the study of aging damage is equivalent to that associated with NAFLD and fibrosis and the different key factors of liver damage such as excessive lipid accumulation, oxidative stress, and liver fibrosis.

Here, we explore the relationship between liver disease with uncontrolled activation of the immune response and natural aging process, which may aggravate liver inflammation and fibrosis. We report that the blockade of the NLRP3 inflammasome attenuates age-associated chronic inflammatory hepatic pathology and thus may be a target for age-related liver disease.

## 2. Materials and Methods

### 2.1. Animal Experimental Design

For all experiments, male mice were used. Young (3 months) and old (20 months) NLRP3−/− (KO) transgenic mice (C57BL/6J background, provided by Bernhard Ryffel and originally generated and characterized in the laboratory of J. Tschopp, [12]) and WT/NLRP3+/+ littermate controls, (*n* = 8) weighing 25–30 g were maintained on a regular 12 h light/dark cycle. All groups had *ad libitum* access to the prescribed diet (13 kcal% fat, 20 kcal% protein, and 67 kcal% carbohydrate, 11.8 kJ/g, Teklad Rodent Maintenance diet 2014S, Harlan Laboratories, Indianapolis, IN, USA) and water throughout the whole study. Mice were housed in a specific facility for a pathogen-free animal, at 20–22 °C with 30–70% relative humidity.

Animal studies were performed in accordance with the European Union guidelines (2010/63/EU) and the corresponding Spanish regulations for the use of laboratory animals in chronic experiments (RD 53/2013 on the care of experimental animals). All experiments were approved by the local institutional animal care committee.

### 2.2. Histological Study

After 20 months, mice were fasted for 12 h and sacrificed to collect the liver. Fasting of mice is necessary to reduce variability in investigatory parameters and to facilitate surgical procedures. Liver tissues were fixed for 12 h with 4% paraformaldehyde, they were included in paraffin and stained with hematoxylin and eosin. Masson’s trichrome staining was used to detect fibrosis in liver sections, and fibrotic areas were also calculated on a digital microscope (×400) with ImageJ (version 1.34S), software (NIH, MD, USA). These mice were compared with young mice (3 months).

Fasting of mice is a common procedure performed in association with many different types of experiments mainly to reduce variability in investigatory parameters or to facilitate surgical procedures.

### 2.3. Standard Biological Parameters

Body weight and food intake were monitored weekly. Serum levels of IGF-1 were assayed in duplicate using commercial ELISA kits (R&D Systems, Minneapolis, MN, USA). Cholesterol and glucose levels were analyzed in duplicate using commercial ELISA kits (Abcam, Cambridge, UK).

### 2.4. Western Blot Analysis

Liver tissue samples of WT and *NLRP3*−/− mice were lysed and homogenized with lysis buffers that included 50 mM HEPES pH 7.5, 5 mM EDTA, 150 mM NaCl, 1% 4-nonylphenyl-polyethylene-glycol, commercial protease inhibitor cocktail (P8340, Sigma-Aldrich, Saint Louis, MO, USA), 1 mM phenylmethylsulfonyl fluoride, 1 mM NaF and 1 mM Na_3_VO_4_. Proteins (50–100 μg) were separated by an Any kD™ Criterion™ TGX Stain-Free™ Protein Gel, 18 well, 30 μL (#5678124, BioRad, Hercules, CA, USA) and transferred to PVDF membranes. The system uses stain-free technology, which is a method that appears to be more reliable as a protein loading control than the measurement of housekeeping proteins. The membranes were incubated with the corresponding commercial primary and secondary antibodies that were coupled to horseradish peroxidase to reveal the protein content by the Clarity™ Western ECL substrate (Ref 170–5061, BioRad, Berkeley, CA, USA) and were analyzed in a ChemiDoc™ Touch Imaging System. Antibodies for the Western blot were obtained commercially and included GATA4 (#36966, Cell Signalling Technology, Danvers, MA, USA), COL1A1 (OABB01299, Aviva Systems Biology, San Diego, CA, USA), TPM2 (#720307, Thermo Fisher Scientific, Waltham, MA, USA), MTTP (ab75316, Abcam, Cambridge, UK), FASN (ab22759, Abcam, Cambridge, UK), DGAT1 (ab54037, Abcam, Cambridge, UK), PTPRG (PA5-15524, Thermo Fisher Scientific, Waltham, MA, USA), p62 (P0067, Sigma Aldrich, Saint Louis, MO, USA), p-mTOR (#5536, Cell Signaling, Beverly, MA, USA), mTOR (#2972, Cell Signaling, Beverly, MA, USA), anti-MAP-LC3 (sc398822, Santa Cruz Biotechnology Inc., Texas, TX, USA), and anti-GAPDH (CB1001, Calbiochem-Merck Chemicals Ldt, Nottingham, UK). The secondary antibody was anti-rabbit (sc-2004, Santa Cruz Biotechnology Inc., Texas, TX, USA) and anti-mouse (sc-2005, Santa Cruz Biotechnology Inc., Texas, TX, USA). The antibody dilutions were 1:1000 for the primary antibodies and 1:10,000 for the secondary antibody. The image analysis was performed using the software Image Lab 6.0 from BioRad (Berkeley, CA, USA).

### 2.5. Oxidative Stress Analyses

SOD activity assay (Canvax, Córdoba, Spain), following manufacturer’s instructions were performed using liver tissue samples of WT and *NLRP3*−/− mice, homogenized and lysed with western blot lysis buffer. The absorbance was measured at 450 nm.

### 2.6. Statistical Analysis

Statistical comparisons among pairs were performed using the unpaired Student’s t-test when the variables were parametric, normally distributed according to Kolmogorov–Smirnov test, and when their variances were homogenous according to Levenne’s F-test. When the variables were nonparametric, the groups were compared using a Mann–Whitney test. The comparisons among groups with parametric variables were tested using one-way ANOVA and a post hoc Tukey test. In the case of nonparametric variables, the Kruskal–Wallis test was used. GraphPad Prism 5.0 (La Jolla, CA, USA) was used to perform all analyses, and all values were expressed as their means ± SEM, where *p*-values less than 0.05 were considered statistically significant.

## 3. Results

### 3.1. The Absence of the NLRP3 Complex Regulates Liver Fibrosis and Biochemical Blood Parameters in Mice

**Figure 1:**
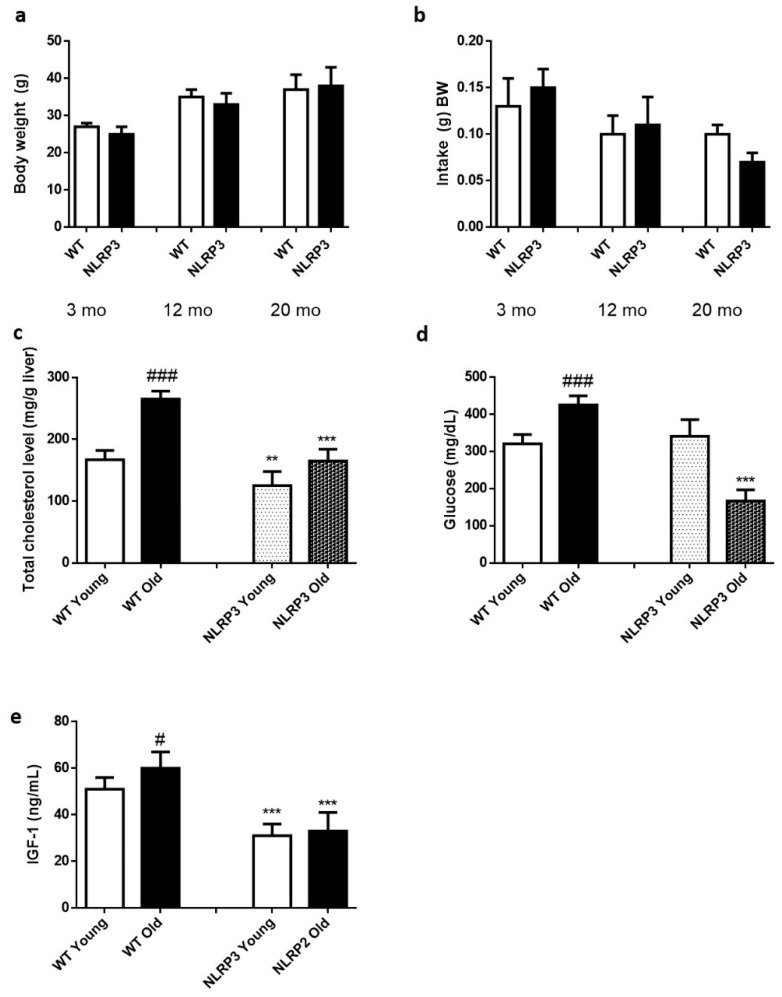
Biochemical parameters of WT and NLRP3−/− mice. (**a**) Changes in body weight along 20 months. (**b**) Food intake (g) BW along 20 months. (**c**) Total cholesterol level (mg/g liver). (**d**) Glucose level (mg/dL). (**e**) IGF-1 levels (ng/mL). All data are shown as the means ± SEM (*n* = 8). ** *p* ≤ 0.01/*** *p* ≤ 0.001 (WT Young/Old vs. NLRP3 Young/Old); # *p* ≤ 0.05/### *p* ≤ 0.001 (WT Young vs. WT old).

The absence of the NLRP3 inflammasome did not generate any change in weight gain over 20 months (Figure 1a,b). Cholesterol levels were significantly reduced in those KO NLRP3 mice (*p* ≤ 0.01) in both young and old mice, compared to their respective controls. However, old mice, both controls and KO NLRP3, had higher cholesterol levels than younger mice (Figure 1c). Glucose levels were significantly reduced (*p* ≤ 0.001) in old KO NLRP3 mice, compared to litter mate controls. In young mice, this trend was not followed, as the absence of NLRP3 complex increased glucose levels, although not significantly (Figure 1d). Finally, IGF-1 levels were significantly reduced (*p* ≤ 0.001) in both groups of mice, young and old KO NLRP3, compared to their respective controls (Figure 1e).

### 3.2. The Inactivation of NLRP3 Inflammasome Complex Expression Decreases the Protein Levels of the Lipid Markers MTTP, FASN, and DGAT1 in Young and Old Mice

**Figure 2:**
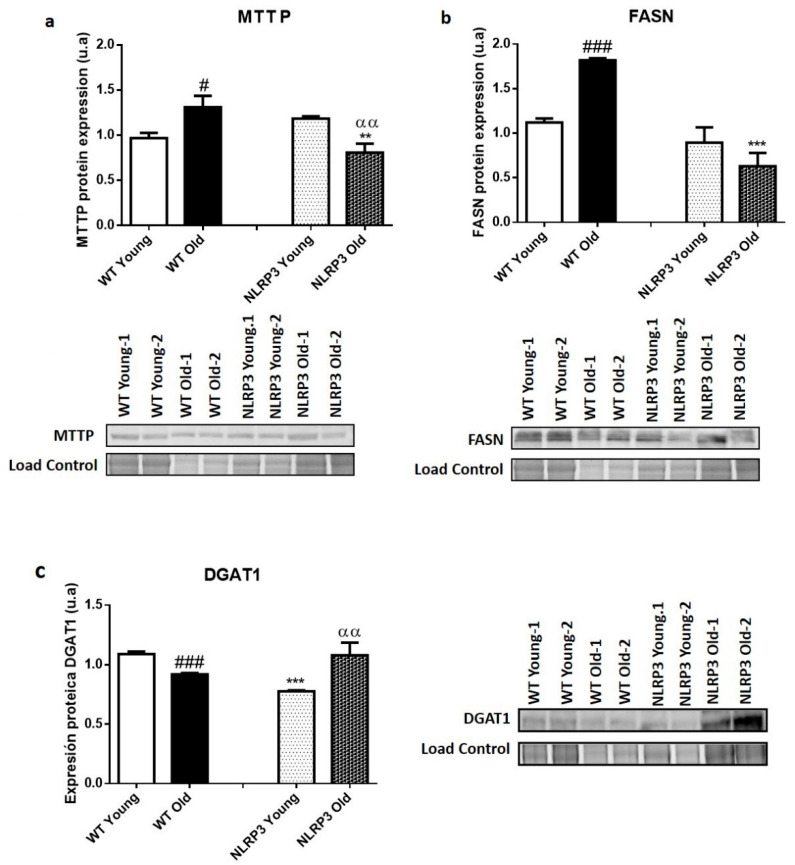
Protein expression levels of lipid metabolism markers in the liver tissue samples of WT and *NLRP3*−/− mice. (**a**) MTTP (**b**) FASN (**c**) DGAT1. All data are shown as the means ± SEM (*n* = 8). ** *p* ≤ 0.01/*** *p* ≤ 0.001 (WT Young/Old vs. NLRP3 Young/Old); # *p* ≤ 0.05/### *p* ≤ 0.001 (WT Young vs. WT old); αα*p* ≤ 0.01(NLRP3 Young vs. NLRP3 old).

Protein expression data in liver tissue showed a significant increase (*p ≤* 0.05) in MTTP protein levels in those older WT mice, compared to younger WT mice (Figure 2a). FASN showed a significant increase (*p ≤* 0.001) in old WT mice, compared to young WT mice (Figure 2b). Finally, DGAT1 showed a different pattern from the rest of lipid metabolism markers, because there was a decrease in DGAT1 levels in the older WT mice, compared with their young WT control (Figure 2c).

Regarding the samples of NLRP3 KO mice, they were found to show significantly reduced levels of the MTTP protein (*p ≤* 0.01) in the case of the old mice compared to their WT control, but not in the case of the young mice, where a non-significant slight increase appear in those mice that did not express NLRP3 compared to young WT. In this case, the effect of the NLRP3 inactivation was higher in the elderly mice, with a significant decrease in the levels of the MTT protein (*p ≤* 0.01) in NLRP3 old mice compared with WT old mice (Figure 2a). FASN was reduced in KO NLRP3 mice of both ages, in comparison with their WT controls, with a significant decrease (*p ≤* 0.001) between older mice (Figure 2b). Finally, the DGAT1 levels only significantly decreased (*p ≤* 0.001) in young KO NLRP3 mice, in comparison with their WT controls. However, the levels of this protein increased in the old mice lacking the inflammasome complex, as compared to their WT controls (Figure 2c).

### 3.3. The Inactivation of NLRP3 Inflammasome Complex Expression Decreases the Levels of Oxidative Stress Markers such as SOD, and of Inflammation as the PTPRG

**Figure 3:**
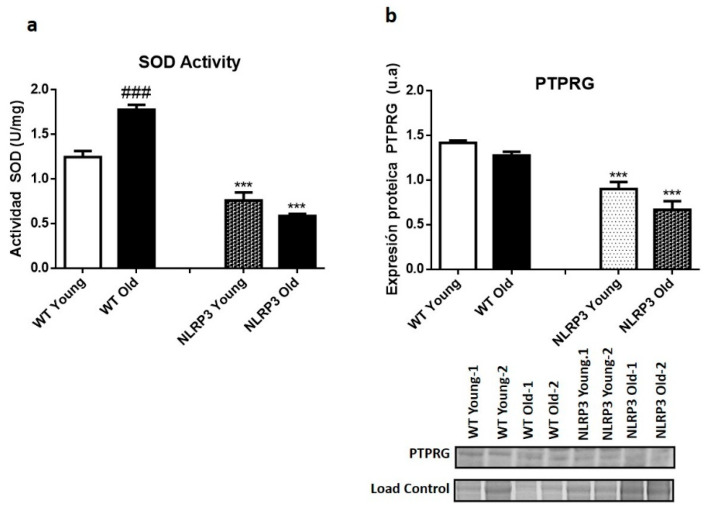
Oxidative stress levels in the liver tissue samples of WT and *NLRP3*−/− mice. (**a**) SOD2 activity. (**b**) Protein levels of PTPRG. All data are shown as the means ± SEM (*n* = 8). *** *p* ≤ 0.001 (WT Young/Old vs. NLRP3 Young/Old); ### *p* ≤ 0.001 (WT Young vs. WT old).

The results of the SOD assay showed a significant decrease in SOD activity (*p ≤* 0.001) in young NLRP3 and old NLRP3 mice samples, compared to their controls. This decrease was somewhat greater in the older mice (Figure 3a). A significant increase (*p ≤* 0.001) in SOD activity occurred in WT old mice compared with their WT young mice controls (Figure 3a). The protein levels of the inflammatory marker PTPRG showed a significant reduction in their levels (*p ≤* 0.001) in those mice, both young and old, KO of the NLRP3 inflammasome complex, in comparison with their WT controls (Figure 3b).

### 3.4. The Absence of the NLRP3 Inflammasome Complex Decreases the Protein Levels of Fibrotic Markers COL-1-α-1 and TPM2β, and Increases the Protective Factor GATA4

**Figure 4:**
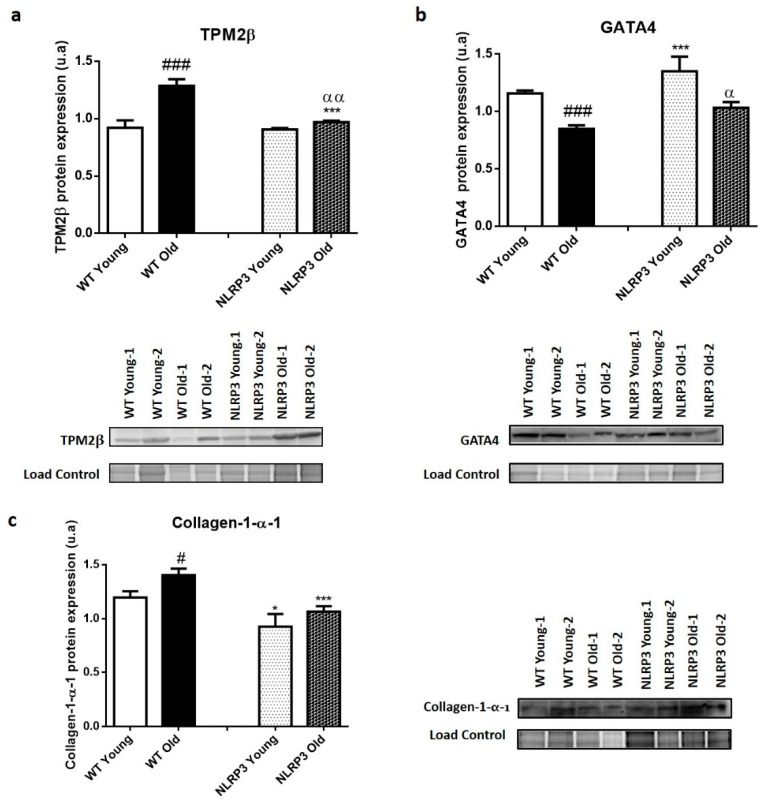
Protein expression levels of fibrotic markers in the liver tissue samples of WT and *NLRP3*−/− mice. (**a**) *TPM2β* (**b**) GATA4 (**c**) Collagen-1-α-1 (COL1α1). All data are shown as the means ± SEM (*n* = 8). * *p* ≤ 0.05/*** *p* ≤ 0.001 (WT Young/Old vs. NLRP3 Young/Old); # *p* ≤ 0.05/### *p* ≤ 0.001 (WT Young vs. WT old); α*p* ≤ 0.05/αα*p* ≤ 0.01 (NLRP3 Young vs. NLRP3 old).

Liver protein expression data revealed a significant increase (*p ≤* 0.001) in the levels of TPM2β (Figure 4a) in old WT mice compared to those young, as well as revealed a significant decrease (*p ≤* 0.001) in the protective factor GATA4 in those WT mice of more advanced ages, in comparison with the mice of early age (Figure 4b). The levels of the Collagen-1-α-1 (COL1α1) fibrotic marker showed a significant increase (*p ≤* 0.05) in WT old mice compared with young WT mice (Figure 4c).

The protein expression data in homogenate liver tissue samples showed similar levels of the TPM2β fibrotic marker in young mice (WT and NLRP3 mice). However, we can observe a significant reduction (*p ≤* 0.001) in NLRP3 old mice compared with WT old mice (Figure 4a); thus, the effect exerted by the inactivation of the NLRP3 complex was more significant in those elderly mice. The levels of the protective factor GATA4 increased in young and old KO mice of the NLRP3 complex, again, showing a significant increase of this marker (*p ≤* 0.001; *p ≤* 0.05) in the old and young mice, respectively (Figure 4b). Finally, the protein levels of COL1α1 decreased when the mice did not express NLRP3, with significant differences (*p ≤* 0.05; *p ≤* 0.001), in young and old mice, respectively (Figure 4c).

**Figure 5:**
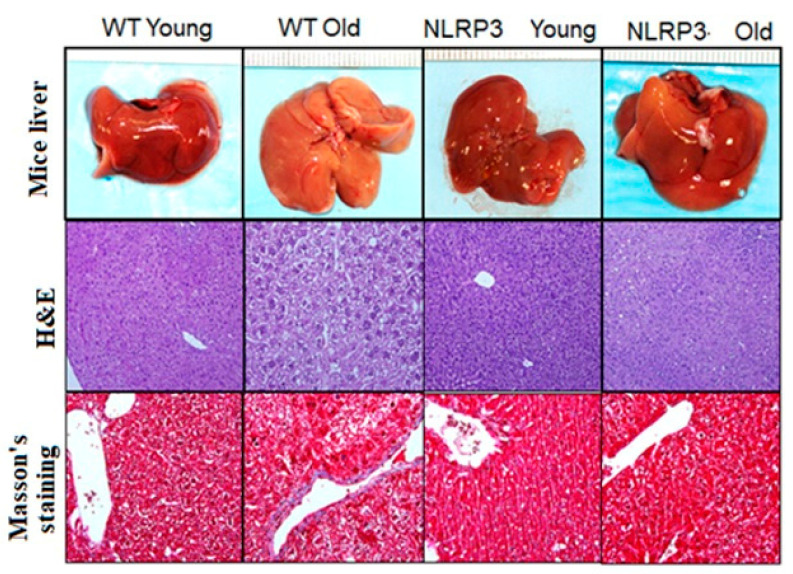
Representative liver sections. Upper: Liver mice; middle: H&E; lower: Masson’s trichrome staining.

H&E staining and Masson’s trichrome staining showed the degree of liver damage (Figure 5). The absence of the NLRP3 inflammasome complex reduced, in both young and old mice, the degree of steatosis compared to WT controls, showing a lower globular deformation of hepatocytes and a lower degree of lobular inflammation. Masson’s staining showed a lower accumulation of type I collagen fibers in those tissue samples from KO NLRP3 mice. The appearance of the livers was normal for all cases.

## 4. Discussion

Aging is a process determined by the progressive loss of tissue and organic function due to the accumulation of reactive oxygen species [9]. Normally, a deterioration of liver function is associated with age, in addition to the appearance of NAFLD, which can progress to liver fibrosis, cirrhosis, and hepatocarcinoma [11]. In this way, lipid accumulation and lipotoxicity, oxidative stress, and inflammation usually play an important role in that progression associated with aging. Liver is responsible for maintaining a metabolic homeostasis in the organism; therefore, an imbalance of the organism due to aging could lead to obesity disorders, such as steatosis, NAFLD, and diabetes [12]. On the other hand, the NLRP3 inflammasome complex, regulator of caspase-1 activation, and maturation of IL1β can be activated by different mediators, including such metabolic alteration [13]. There are different studies that ensure the relationship between the NLRP3 inflammasome complex and liver lesions, increasing the expression of liver NLRP3 and caspase-1, and finally generating an inflammatory process in the liver [7,8].

Recent studies have shown that the inflammasome complex NLRP3 in liver stellate cells can directly regulate its activation and contribute to liver fibrosis [14]. In addition to this, other studies have demonstrated the relationship between lipotoxicity and the activation of the NLRP3 complex in liver cells. Specifically, microvesicles released from fat-laden liver cells promoted the activation of the NLRP3 inflammasome, with a pro-inflammatory link between lipotoxicity and non-alcoholic steatohepatitis [15]. These results suggest for studies with the whole livers of KO NLRP3 mice to elucidate the relationship between the inflammatory process and the damage associated with NAFLD, such as lipid accumulation, oxidation, and liver fibrosis.

### 4.1. Regulation of Hepatic Lipid Metabolism by NLRP3 Suppression in Aging Mice

To know the metabolic state in which the different control groups of young and old mice were, cholesterol and blood glucose levels were measured. As expected, and due to the aging process, that group of old mice showed higher levels of cholesterol and blood glucose compared to the young control group. However, and repairing in the KO NLRP3 mice, a significant decrease in cholesterol and glucose levels was observed compared to the control groups. This result provides a clear indication of the relationship between the inflammatory process and the metabolic state of the organism, laying the foundations to deepen a study in which the suppression of the NLRP3 complex in young and old mice would help us know in what state of activation are the different lipid, oxidative, and fibrotic markers.

Microsomal triglyceride transfer protein (MTP) is a protein with the ability to transfer lipids between different membrane vesicles, in addition to being involved in the regulation of cholesterol synthesis [16]. MTP levels decreased in KO NLRP3 mice, with a greater difference from the control group. This trend was repeated with another metabolic marker, fatty acid synthase (FASN), involved in the biosynthesis of fatty acids. On the one hand, this protein expression reached significantly high levels in the WT old group compared to the WT young group. These data agree with those of a recent investigation in which different groups of mice in a progressive state of aging increased their levels of fatty acids and triglycerides in parallel to the older age [17]. As for the groups of KO NLRP3 mice, especially the group of old mice, there was a significantly reduced FASN protein levels compared to the control group. These results confirm the relationship between lipid metabolism and inflammatory process. Finally, in the analysis of the last metabolic marker, diacylglycerol acyl transferase (DGAT1), the results were somewhat different. Unlike the markers previously studied, DGAT1 protein levels were only reduced in those young KO NLRP3 mice, while, in old mice, the levels were higher in the KO NLRP3 group compared to control groups. It is possible to say that, despite this result, the differences between old mice groups were not significant, staying within levels of protein expression that slightly surpassed the control. The aging process is usually associated with an increase in DGAT1 levels, and a deficiency of this is related to greater longevity [18]. It is possible that the increase of DGAT1 levels associated with aging could be so high that the suppression of NLRP3 cannot reduce them below the control.

### 4.2. Regulation of Hepatic Oxidative Stress by NLRP3 Suppression in Aging Mice

The activation of inflammasome after damage due to excessive lipid accumulation and metabolic alteration is usually accompanied by the release of reactive oxygen species. At the same time, oxidative stress is associated with the aging process, producing an infiltration of neutrophils and a generation of chemokines that, finally, activate hepatic stellate cells, resulting in a fibrosis process [19]. In the analysis of the oxidative stress markers, SOD activity increased significantly, as expected, in that control group of WT old mice, compared to the control group of young mice. The suppression of the NLRP3 inflammasome complex reduced these levels in both groups of mice, and this decrease was more pronounced in the group of old mice. The PTPRG marker, another oxidative stress marker, reduced protein levels in those groups of KO NLRP3 mice with advanced age in a significant way compared to their WT old control. All this is finally related to the fibrotic state of the liver.

### 4.3. Regulation of Hepatic Fibrosis by NLRP3 Suppression in Aging Mice

Liver damage associated with lipid accumulation and oxidative stress is related to the activation of hepatic stellate cells, stating a fibrotic process in the liver. Aging affects liver pathology, aggravating symptoms and changes occurring at molecular level. In fact, a recent study, in which elderly rats treated with simvastatin or PBS in those controls, resulted in a severe form of advanced chronic liver disease, exacerbating portal hypertension and hepatic fibrosis with increased hepatocyte cell death and poorer function, in the aged control animals. In addition, hepatic stellate cells were activated and there was a macrophage infiltration that aggravated the disease [20]. These data explain that in our elderly control mice, the protein levels of the COL1α1 fibrotic marker increased compared to those in which the NLRP3 complex was suppressed. A similar result is shown in Masson’s trichrome staining. In this experiment, the old control mice showed accumulation of blue-stained collagen fibers, while in those KO NLRP3 Old mice, collagen fibers deposition was reduced. These data are supported by a study in which mice with activated NLRP3 showed greater hepatic steatosis and fibrosis, while suppression of NLRP3 complex was protective against the development of these liver conditions, accompanied by a lower macrophage infiltration [3]. The analysis of the fibrotic marker TPM2β in control mice and KO NLRP3 resulted in a reduction of their protein levels in those old mice in which the inflammasome complex had been suppressed, compared to their controls. Similarly, the protective factor GATA4 in KO NLRP3 mice increased, in both young and old mice, above their controls, indicating the protective role that suppression of the NLRP3 inflammasome complex presented to liver damage due to fibrosis.

## 5. Conclusions

The suppression of the NLRP3 complex is protective against damage related to liver diseases such as NAFLD, which are frequent and are aggravated by the aging process. Thus, its suppression could reduce markers of lipid metabolism and oxidative stress, improving the fibrotic state of liver cells in those KO mice, observing better results in those old mice. The underlying mechanisms that explain these results are still unclear and further investigation is required. One possible way would be the study of the expression levels of lipid markers such as perilipins 2 (plin2) and perilipins 3 (plin 3). Reducing the expression levels of these experiments in live experiments protects animal models from induced metabolic diseases. Experimental data suggested that, at least in NAFLD, a possible link between plin2/plin3 and hepatic steatosis could be inflammation. Furthermore, the expression of these proteins appears associated with pathological conditions and age-related diseases [21,22]. That is why the study of the expression of these markers could help to elucidate the molecular mechanisms underlying the reduction of lipid, fibrotic, and oxidative levels that occur in those KO NLRP3 mice, which could be affecting perilipins.

In conclusion, we can affirm the role of the NLRP3 inflammasome complex in the progression of liver diseases. The regulation of the NLRP3 complex could become a new alternative to the treatment of NAFLD and liver fibrosis, especially in elderly people with severe liver damage.

## Abbreviations

ASC (apoptosis-associated speck-like protein to CARD), COL1α1 (collagen1α1), DGAT1 (diacylglycerol o-acyltransferase 1), FAS (fatty acid synthase), HSCs (hepatic stellate cells), iNOS (inducible nitric oxide synthase), KO (knockout), MTTP (microsomal triglyceride transfer protein), NAFLD (non-alcoholic fatty liver disease), NOD (NOD-type receptor), PTPRG (receptor-type tyrosine-protein phosphatase gamma), SOD (superoxide dismutase), TNF-α (tumor necrosis factor-α), TPM2β (tropomyosin 2β).
